# Anti-interferon-γ autoantibodies: immunopathogenesis, diagnostic innovation, and emerging therapeutic strategies in an adult-onset immunodeficiency

**DOI:** 10.3389/fimmu.2026.1964087

**Published:** 2026-09-30

**Authors:** Chin-Shiang Tsai, Aristine Cheng, Farha Naz

**Affiliations:** 1 Department of Internal Medicine, National Cheng Kung University Hospital, College of Medicine, National Cheng Kung University, Tainan, Taiwan; 2 Division of Infectious Diseases and International Health, Department of Medicine, University of Virginia School of Medicine, Charlottesville, VA, United States; 3 Department of Internal Medicine, National Taiwan University Hospital and National Taiwan University College of Medicine, Taipei, Taiwan

**Keywords:** adult-onset immunodeficiency, anti-interferon-γ autoantibodies, clinical decision pathway, HLA susceptibility, nontuberculous mycobacteria, rituximab

## Abstract

Adult-onset immunodeficiency with anti–interferon-γ autoantibodies (AIGA) is a rare acquired immunodeficiency increasingly recognized in East and Southeast Asia, characterized by disseminated infections with nontuberculous mycobacteria and other intracellular pathogens. Recent studies have refined our understanding of its immunopathogenesis, including HLA-linked susceptibility, epitope-specific neutralization of IFN-γ, and molecular mimicry, while highlighting persistent gaps in diagnosis and treatment. This Perspective synthesizes emerging evidence into a clinically oriented framework spanning diagnosis, monitoring, and immunomodulatory therapy. We discuss functional confirmation of AIGA, repurposing of the QuantiFERON-TB platform as an accessible screening strategy, and quantitative titers and ¹^8^F-FDG PET as complementary biomarkers of disease activity and treatment response. We propose a clinical decision pathway in which refractory or progressive infection, rather than a fixed titer threshold, triggers immunomodulation, with rituximab and cyclophosphamide as the best-supported induction strategies, daratumumab reserved for refractory cases, and emerging AIGA-specific evidence supporting azathioprine for maintenance. Recognizing heterogeneous access to specialized testing and biologics across AIGA-endemic regions, we propose a resource-stratified approach integrating widely available markers with referral-based testing and resource-adapted treatment. We also highlight emerging antigen-specific strategies, including chimeric autoantibody receptor T cells engineered to eliminate autoantibody-producing B cells, as a future direction toward durable remission. Finally, we outline priorities for prospective validation and biomarker-guided therapy.

## Introduction

Anti-interferon-γ autoantibodies (AIGA) cause an acquired, adult-onset syndrome that functionally phenocopies Mendelian susceptibility to mycobacterial disease (1). The syndrome disrupts the same interferon-γ (IFN-γ)-signal transducer and activator of transcription 1 (STAT1)-macrophage activation axis that monogenic interleukin-12 (IL-12)/IFN-γ pathway defects disrupt congenitally, but it does so through autoimmunity rather than germline mutation (2). First described independently by several groups between 2004 and 2005 (3–5), AIGA is now recognized as a leading cause of disseminated, unexplained opportunistic infection, most commonly nontuberculous mycobacteria (NTM) but also *Talaromyces marneffei*, nontyphoidal *Salmonella*, and reactivated varicella-zoster virus (VZV), predominantly in human leukocyte antigen (HLA)-susceptible adults across East and Southeast Asia. AIGA is also increasingly relevant outside endemic regions, including the United States, particularly among patients of Southeast Asian ancestry. Comparative natural history data from Thailand and a National Institutes of Health (NIH) cohort demonstrated distinct demographic, microbiologic, and clinical features between the two settings, suggesting that disease expression may reflect not only genetic susceptibility but also geography, environmental exposure, and other host or healthcare-related factors (6).

The immunopathogenesis of AIGA, including its HLA associations, epitope specificity, and downstream signaling defects, has been comprehensively reviewed (7), and a broader mechanistic framework encompassing anti-cytokine autoantibodies across many diseases, including AIGA, has recently been proposed (8). These reviews provide a comprehensive mechanistic foundation on which further clinical translation can build. What remains comparatively underdeveloped is the translation of this mechanistic understanding into a clinical framework that a treating physician can act on: when to escalate beyond antimicrobial therapy, which immunomodulatory agent to select and on what basis, and which of the available strategies rest on AIGA-specific evidence rather than extrapolation from other antibody-mediated diseases.

Here, we attempt to bridge that gap by proposing a clinical framework grounded in AIGA-specific evidence. AIGA immunopathogenesis is summarized only insofar as it motivates diagnostic and therapeutic decisions; diagnostic tools are reviewed across a spectrum of clinical accessibility; a clinical decision pathway linking baseline assessment to treatment is proposed; and specific research directions likely to improve patient outcomes are identified.

## Immunopathogenesis

AIGA disrupts IFN-γ signaling through a partially-characterized mechanism. HLA-DRB1*16:02 and HLA-DQB1*05:02, or the related HLA-DRB1*15:02/HLA-DQB1*05:01 haplotype, are present in 80–90% of affected patients and confer strong genetic susceptibility across Thailand, Taiwan, South China, and Hong Kong, with sporadic cases reported elsewhere in Southeast Asia (9–11). AIGA is also encountered in the United States, particularly among individuals of Southeast Asian ancestry; in an NIH cohort, 91% of US patients were of this background, though their phenotype differed from Thai patients in predominant NTM species and sites of infection (6), suggesting that geography and host factors shape disease expression beyond genetic ancestry alone.

Within this population, autoantibodies are polyclonal, targeting both linear (a conserved C-terminal region, residues 121-143, including the KRKR motif at 127-131) and conformational epitopes of IFN-γ, blocking receptor engagement and STAT1 phosphorylation (12), thereby impeding macrophage activation and the IL-12/IFN-γ feedback loop (7). Molecular mimicry with the Aspergillus *Noc2* ribosome assembly protein is one proposed trigger for this loss of tolerance (12), though it does not explain why disease develops in only a subset of HLA-susceptible, *Aspergillus*-exposed individuals, an open question addressed below.

This mechanism directly motivates two clinical tools discussed in the following sections: it explains why neutralizing titer, rather than binding titer alone, determines pathogenicity, and why therapies that deplete antibody-producing cells, rather than only replacing IFN-γ, form the basis of current treatment.

## Diagnostic innovation: from research assay to clinical accessibility

A confirmed AIGA diagnosis requires both detecting the autoantibody and demonstrating its neutralizing activity, since binding alone is not necessarily pathogenic (13). The most rigorous functional assay, flow cytometric quantification of IFN-γ-induced STAT1 phosphorylation, is confined to research and specialized centers (3), as summarized in **Table 1**; this is particularly relevant in the United States and other settings where AIGA is uncommon and validated testing may require referral. Repurposing the widely available QuantiFERON-TB platform as a functional screening tool has therefore been explored and validated independently (14, 15). The assay’s mitogen tube stimulates lymphocytes with phytohemagglutinin to produce IFN-γ, quantified by ELISA; in neutralizing AIGA, this response becomes extremely low or undetectable since the IFN-γ released is bound by the polyclonal high affinity autoantibodies, yielding an indeterminate result (14, 15). Spiking AIGA-containing serum into healthy donor blood similarly abrogated the mitogen response, confirming that the inhibitory activity resides in the serum itself rather than impaired T-cell function (15). Because QuantiFERON is already widely deployed clinically, this could provide accessible functional screening where dedicated AIGA testing is unavailable, though its use remains nonstandard and should not substitute for confirmatory testing when available.

**Table 1:** Diagnostic tools and immunomodulatory agents in AIGA, by role and evidentiary basis.

Tool/agent	Role	Clinical availability/evidence level	Reference
Diagnostic tools			
Serum IgG, CD19^+^ B cell count	Baseline immune assessment; monitoring B-cell depletion	Routinely available	(21)
Neutralizing AIGA titer (functional assay)	Diagnostic confirmation; treatment-response monitoring	Specialized/research laboratory; limited routine clinical availability	(3, 16)
QuantiFERON-TB (repurposed)	Functional screening	Platform widely available; AIGA application nonstandard	(14, 15)
Whole-body ^18^F-FDG PET	Disease-burden mapping; identification of occult foci; outcome prediction	Clinically available; selective use, with access/reimbursement dependent on indication	(18)
Induction therapy			
Rituximab	Induction option; largest evidence base	Clinically available; AIGA-specific observational evidence	(19, 21, 22)
Cyclophosphamide	Alternative/adjunct induction	Clinically available; AIGA-specific observational evidence	(16)
Daratumumab	Salvage therapy for refractory disease	Clinically available; AIGA evidence limited to a single case	(23)
Immunoablation/autologous HSCT	Experimental/theoretical salvage strategy	Specialized transplant centers; no reported AIGA experience	(24)
Maintenance therapy (post-induction)			
Azathioprine	Maintenance option	Routinely available; AIGA-specific observational evidence	(33)
Methotrexate	Alternative maintenance candidate	Routinely available; no AIGA-specific evidence; extrapolated from ANCA-associated vasculitis	(34)
Belimumab	Proposed maintenance	AIGA-specific evidence limited to an unpublished case series; otherwise extrapolated from antibody-mediated autoimmune disease	(43–46)

AIGA, anti-interferon-γ autoantibody; ANCA, antineutrophil cytoplasmic antibody; HSCT, hematopoietic stem cell transplantation; PET, positron emission tomography; SLE, systemic lupus erythematosus. Clinical availability/evidence level reflects the current accessibility of each diagnostic or therapeutic approach and the strength of published evidence specifically supporting its use in AIGA; availability may vary across institutions and healthcare systems. These categories do not indicate diagnostic accuracy, comparative efficacy, or formal guideline endorsement. Numbers in parentheses refer to the reference list.

Beyond diagnosis, quantitative AIGA titers may serve as treatment-response biomarkers, though no universal threshold defines a clinically significant titer, since values are assay-dependent and not interchangeable across platforms. In a cyclophosphamide cohort, patients achieving titer reduction to approximately 5,000 by six months had significantly better outcomes than those remaining near 100,000, from an initial median around 200,000 (16). This distinction should be interpreted cautiously: these assays measure binding titer as a rough proxy for neutralizing activity and have not been validated for monitoring individual patient responses. In a bortezomib trial using a distinct ELISA platform in arbitrary units, baseline titers had a median of 6,370.1 AU (IQR 592.1–22,464.8), with no significant reduction at 8 or 48 weeks (17). These values should therefore be viewed as study-specific reference ranges rather than interchangeable clinical thresholds.

Whole-body ¹^8^F-fluorodeoxyglucose positron emission tomography (¹^8^F-FDG PET) has also demonstrated utility, identifying additional infectious foci beyond conventional evaluation in most patients in one cohort, with splenic involvement independently predicting rehospitalization within one year (18), informing both antimicrobial extent and the baseline for later response assessment.

A limitation cuts across all of these tools: the evidence base is heavily weighted toward NTM-associated disease. *Talaromyces marneffei*, nontyphoidal *Salmonella*, and VZV reactivation, while well documented as part of the AIGA pathogen spectrum (19), have not been the focus of dedicated diagnostic validation studies. Whether titer thresholds, PET uptake patterns, or treatment response kinetics established in NTM cohorts generalize to these other pathogens is untested and represents a specific, addressable gap, particularly since several of these pathogens are, if anything, more acutely life-threatening at presentation than NTM (20).

## A proposed clinical decision pathway for immunomodulation

Pathogen-directed antimicrobial therapy remains the non-negotiable foundation of AIGA management regardless of subsequent immunomodulatory decisions. The more difficult clinical question is when, and with what agent, to additionally target the underlying autoantibody-producing process. We propose a decision pathway (**Figure 1**) built on published AIGA-specific evidence.

**Figure 1:**
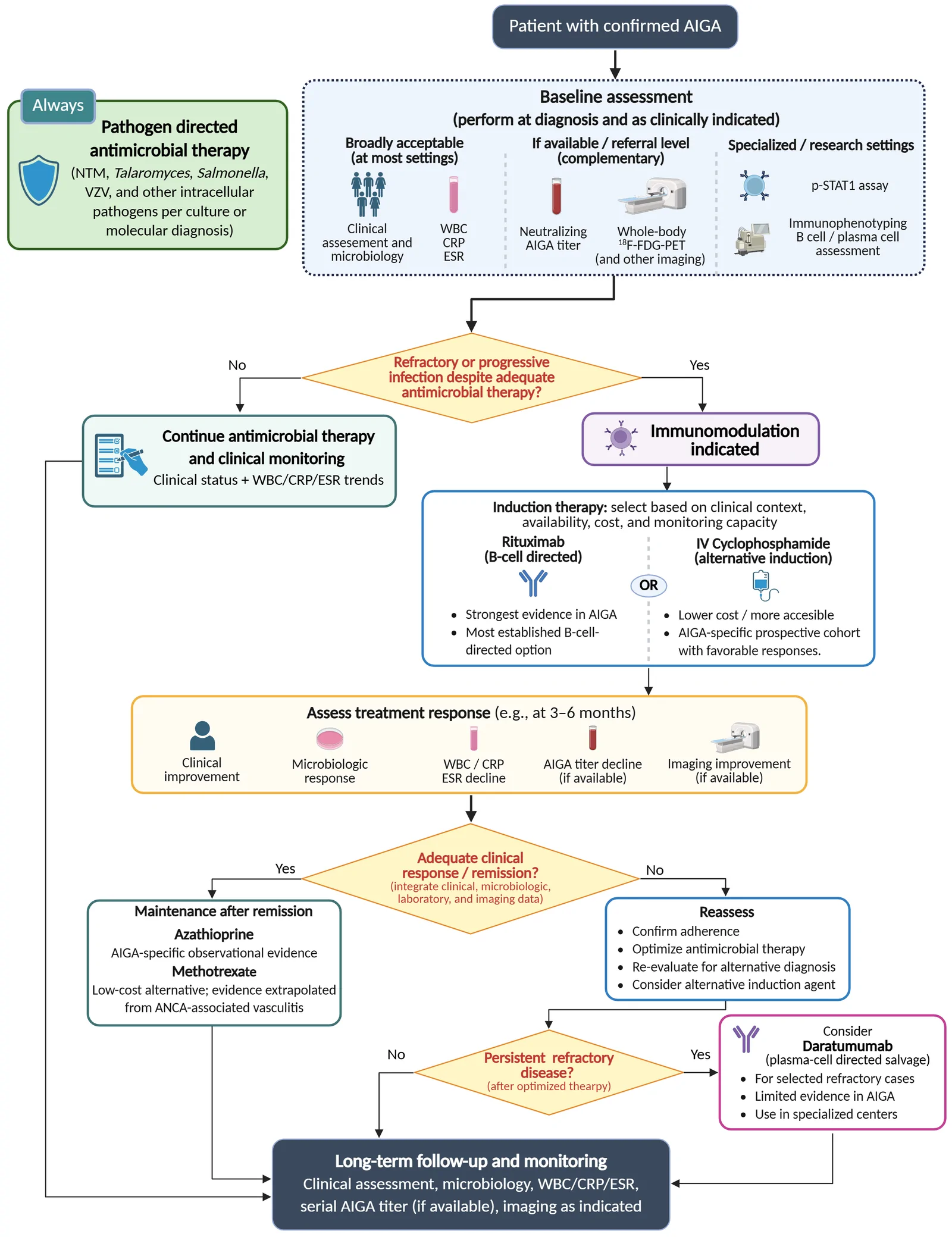
Proposed clinical decision pathway for treatment selection and longitudinal monitoring in adult-onset immunodeficiency with anti–interferon-γ autoantibodies (AIGA). Pathogen-directed antimicrobial therapy remains the foundation of management throughout the disease course. Baseline and response assessments integrate clinical and microbiologic evaluation with widely available inflammatory markers, while neutralizing AIGA titers, ^18^F-fluorodeoxyglucose positron emission tomography (^18^F-FDG PET), STAT1 phosphorylation assays, and detailed immunophenotyping are incorporated according to availability and clinical need. Refractory or progressive infection despite adequate antimicrobial therapy, rather than a predefined AIGA titer threshold, serves as the principal trigger for immunomodulation. Rituximab and intravenous cyclophosphamide are presented as induction options, with selection individualized according to clinical context, availability, cost, and monitoring capacity. Following remission, azathioprine has AIGA-specific observational evidence as maintenance therapy, whereas methotrexate represents a lower-cost candidate supported by extrapolation from other antibody-mediated diseases. Daratumumab is reserved as a plasma-cell-directed salvage option for selected patients with persistent refractory disease after optimized therapy. All pathways converge on longitudinal clinical, microbiologic, and biomarker monitoring. AIGA, anti–interferon-γ autoantibodies; CRP, C-reactive protein; ESR, erythrocyte sedimentation rate; FDG, fluorodeoxyglucose; NTM, nontuberculous mycobacteria; PET, positron emission tomography; pSTAT1, phosphorylated signal transducer and activator of transcription 1; VZV, varicella-zoster virus; WBC, white blood cell count.

The central gatekeeping decision is whether infection remains refractory or progressive despite adequate antimicrobial therapy. Neutralizing AIGA titers are best interpreted as a complementary biomarker rather than a fixed threshold, since no cutoff has been validated across assay platforms; serial within-patient trends, rather than absolute values, are more informative, with declining titers associated with favorable response (16, 19, 21). Among patients meeting this threshold, rituximab and intravenous cyclophosphamide represent the two best-supported induction options, with selection individualized by context, availability, cost, and monitoring capacity rather than a fixed hierarchy. Rituximab has the largest evidence base, with CD20^+^ B cell depletion accompanied by titer decline and restored IFN-γ-STAT1 signaling in published case series (19, 21, 22). Cyclophosphamide offers a mechanistically distinct, lower-cost alternative supported by a small prospective AIGA cohort; in that cohort, titer reduction to a median of approximately 5,000 by two years was observed, with titer levels at six months already differing between eventual responders and non-responders (16). Because evidence for both agents remains limited to small, non-randomized cohorts, the choice should be guided by resource availability rather than comparative superiority.

Daratumumab, targeting CD38^+^ cells including long-lived plasma cells that are largely unreachable by rituximab, has been reported as salvage therapy after rituximab failure in a single case, with accompanying decline in both titer and total IgG (23). For the small subset of patients with disease refractory to both approaches, immunoablation followed by autologous hematopoietic stem cell transplantation has been used in other refractory autoimmune diseases (24) and represents a theoretical option, though it carries substantial infectious risk in a population already defined by impaired antimicrobial defense, and has not, to our knowledge, been reported in AIGA specifically. Regardless of the induction agent chosen, long-term follow-up combining clinical assessment with serial biomarkers, detailed further below, remains necessary to confirm sustained response and detect early signs of relapse before clinical deterioration occurs (7, 16).

Safety monitoring and infection prophylaxis. Immunomodulatory therapy adds treatment-related infection risk to the underlying impairment of antimicrobial defense in AIGA. Before rituximab initiation, screening for hepatitis B virus with HBsAg, anti-HBc, and anti-HBs is warranted, with antiviral prophylaxis recommended for patients with chronic HBV infection and for those with past infection receiving high-risk B-cell-depleting therapy (25, 26). In the absence of AIGA-specific data, *Pneumocystis jirovecii* pneumonia prophylaxis should be considered according to the intensity and duration of immunosuppression; guidance from ANCA-associated vasculitis supports trimethoprim-sulfamethoxazole prophylaxis in patients receiving rituximab, cyclophosphamide, and/or high-dose glucocorticoids (27). Serial CD19^+^ B-cell counts may help document B-cell depletion and repopulation after rituximab (21). Cyclophosphamide additionally requires hematologic monitoring and surveillance for urotoxicity (27), whereas daratumumab may exacerbate hypogammaglobulinemia, warranting serial immunoglobulin measurement (28).

## Resource-stratified diagnosis and treatment of AIGA

Because AIGA is concentrated in East and Southeast Asia, where access to specialized testing and biologics varies substantially, a practical pathway should distinguish essential care from resource-dependent adjuncts. At all levels, recognition of the clinical phenotype and microbiologic identification of pathogens remain foundational, and antimicrobial therapy should not be delayed awaiting specialized testing. Where neutralization assays are unavailable, a markedly suppressed QuantiFERON-TB mitogen response can provide an accessible clue justifying referral for confirmatory testing (14, 15); commercially available QuantiFERON reagents have also been adapted for quantitative inhibitory testing, a scalable option where dedicated platforms are unavailable (29).

Resource constraints are equally relevant to monitoring and treatment. When serial titers or advanced imaging are unavailable, clinical assessment combined with widely available markers (WBC, CRP, ESR) can provide pragmatic measures of activity; these consistently decline with remission, and a combined panel retains useful discriminatory performance even without titers (30, 31). Specialized centers can add serial titers, STAT1 phosphorylation assays, PET, and detailed immunophenotyping when likely to alter management. Treatment should follow the same principle: rituximab remains the best-supported B-cell-directed therapy, but intravenous cyclophosphamide represents a lower-cost, more accessible alternative where rituximab is unavailable, supported by a small prospective cohort with favorable responses (32); because this evidence remains limited and nonrandomized, availability should guide choice without implying equivalence. For remission maintenance, azathioprine is an inexpensive option supported by recent AIGA-specific data showing prolonged relapse-free survival (33), while methotrexate is another inexpensive candidate, though its evidence is extrapolated from ANCA-associated vasculitis (34). Daratumumab should remain reserved for refractory cases in centers able to provide the required monitoring.

## Directions for future research

Several strategies with no direct AIGA-specific evidence base have nonetheless demonstrated proof-of-concept in mechanistically related antibody-mediated diseases, and a broader menu of ACAA-directed approaches, including Treg modulation and T cell co-stimulation blockade, has recently been proposed at the level of the entire field (8). We highlight five directions where AIGA-specific investigation would most directly change patient management.

First, baseline immunophenotyping. Whether flow cytometric quantification of CD20+ B cells versus CD38+CD20- plasma cells before treatment could identify patients unlikely to respond to rituximab, sparing a trial of B cell depletion before a plasma cell-directed agent, is untested in AIGA and speculative even by precedent: baseline subset enumeration alone predicts rituximab response poorly in rheumatoid arthritis (35), where post-treatment depletion depth is more informative (36). Precedent from refractory SLE, where daratumumab induced responses in patients whose plasma cells were unreachable by rituximab, motivates this as testable rather than validated. A retrospective cohort with paired baseline flow cytometry and outcome would suffice to test it.

Second, deeper phenotypic and clonal characterization of AIGA-producing B-cell and plasma-cell populations. CD138, CD27, and CD38 are incorporated into standardized panels for plasma-cell disorders (37), but not for AIGA; adding these to the CD20/CD38 framework above could better distinguish B-cell, plasmablast, and plasma-cell populations relevant to treatment. BCR repertoire and single-cell sequencing could identify clonally expanded populations linked to disease activity or relapse; however, clonal expansion alone would not establish pathogenicity, and functional testing of clone-derived antibodies for neutralization would be required (38). Precedent links CD27+CD38hi populations to relapse in ANCA-associated vasculitis (39) and BCR dynamics to rituximab response in rheumatoid arthritis (40).

Third, antigen-specific cellular therapy building on this characterization. Chimeric autoantibody receptor (CAAR) T cells, engineered with a receptor-unresponsive IFN-γ bait recognized by AIGA-producing B cell receptors, eliminate autoantibody-producing B cells in a humanized mouse model and *ex vivo* patient samples without cross-reactivity against the ubiquitous IFN-γ receptor (41). This strategy is generalizable to other ACAAs and offers durable, antigen-specific remission, though efficacy against long-lived plasma cells remains open, possibly requiring co-deployment with plasma-cell-directed agents. A mechanistically distinct approach, neonatal Fc receptor (FcRn) inhibition, accelerates catabolism of pathogenic IgG and has shown efficacy in other antibody-mediated diseases (42); however, FcRn blockade is antigen-agnostic and would nonselectively deplete the protective IgG that AIGA patients depend on for infection control, limiting its appeal in this population without concurrent antimicrobial and immunoglobulin support.

Fourth, a multicenter prospective registry linking baseline titer, immunophenotype, and pathogen at presentation to treatment choice and outcome. Nearly all current evidence, including the pathway proposed here, derives from retrospective series concentrated in a small number of centers (19). A registry designed prospectively around the decision points in Figure would allow the pathway to be validated or revised with actual outcome data, rather than through further retrospective description.

Fifth, identifying the environmental or infectious trigger that converts HLA susceptibility into overt autoimmunity. Molecular mimicry with the *Aspergillus* Noc2 protein provides experimental proof of concept (12), but exposure to this ubiquitous fungal genus is far more common than AIGA itself, even among HLA-susceptible individuals, implying that exposure alone is insufficient to explain disease onset. A prospective cohort following HLA-susceptible, currently healthy individuals in endemic regions, with serial serologic and environmental exposure sampling preceding any seroconversion to AIGA positivity, would offer the most direct route to identifying what distinguishes those who progress to disease from those who do not. Were such a second hit identified, it would raise the possibility of preventing AIGA before it develops, a possibility not available for any of the treatment-stage strategies discussed above.

Maintenance therapy following induction of remission remains an important unresolved component of AIGA management, particularly because recurrence after treatment discontinuation has been associated with resurgence of AIGA titers (7). Recent AIGA-specific evidence has begun to address this gap. In a retrospective Thai cohort, azathioprine maintenance was associated with substantially prolonged relapse-free survival compared with no maintenance therapy (median 3.99 versus 0.88 years), with an adjusted hazard ratio for relapse of 0.096 (33). Although limited by its retrospective, single-center design and small sample size, this study provides direct clinical evidence supporting azathioprine as a potential maintenance strategy in AIGA. Beyond azathioprine, other maintenance approaches remain largely extrapolated from antibody-mediated diseases. Belimumab, administered sequentially after rituximab, can attenuate the compensatory surge in B cell-activating factor (BAFF) following B-cell depletion (43), with two-year follow-up demonstrating sustained low disease activity in the majority of treated patients with severe, refractory systemic lupus erythematosus (SLE) (44), and has been used as maintenance therapy in a case series of AIGA patients (Weng et al., unpublished data).

A similarly designed randomized trial demonstrated that sequential belimumab and rituximab enhanced salivary gland B cell depletion and improved disease activity scores relative to either agent alone in primary Sjögren’s syndrome (45). A comparable trial in antineutrophil cytoplasmic antibody (ANCA)-associated vasculitis has completed enrollment, though results have not yet been published (46). Methotrexate has separately demonstrated comparable relapse rates to azathioprine as maintenance therapy following cyclophosphamide-induced remission in ANCA-associated vasculitis (34), another B cell/autoantibody-mediated disease with an induction-relapse pattern comparable to AIGA. Because AIGA shares this same fundamental problem, that is, resurgence of pathogenic autoantibody-producing cells following induction, both sequential belimumab after rituximab and methotrexate maintenance represent mechanistically motivated, testable candidates for this unaddressed component of AIGA management, though neither has been evaluated in AIGA specifically.

## Conclusion

AIGA-associated immunodeficiency occupies a distinctive position at the intersection of acquired autoimmunity and infectious disease susceptibility, a syndrome in which the underlying defect, unlike its monogenic phenocopies, is in principle reversible. The immunopathogenesis is slowly being characterized, and diagnostic tools are becoming progressively more accessible. Emerging AIGA-specific evidence, including support for azathioprine as maintenance therapy, is beginning to close gaps in this pathway. What remains missing is a fully validated, evidence-graded pathway linking baseline assessment to treatment selection, together with prospective data testing the hypotheses proposed here: immunophenotype-guided therapy selection, clonal and antigen-specific cellular approaches to eliminating pathogenic B cell and plasma cell populations, identification of the environmental trigger converting genetic susceptibility to disease, and registry-based outcome tracking. Given the geographic concentration of AIGA in East and Southeast Asia but its recognition in the United States and other non-endemic settings, international prospective cohorts will be important for determining which features of disease and treatment response are shared across populations and which are context dependent. Testing these hypotheses, alongside continued accumulation of AIGA-specific evidence, represents the most direct path toward evidence-based AIGA management.

## Data Availability

The original contributions presented in the study are included in the article/supplementary material. Further inquiries can be directed to the corresponding author.
